# Hsa_circ_0001944 enhanced GSPT1 expression via sponging miR‐498 to promote proliferation and invasion of gastric cancer

**DOI:** 10.1002/jcla.24810

**Published:** 2023-01-04

**Authors:** Rujiao Liu, Xiaotian Han, Shuiping Gao, Yang Chen, Jian Zhang

**Affiliations:** ^1^ Phase I Clinical Trial Center Fudan University Shanghai Cancer Center Shanghai China; ^2^ Department of Oncology Shanghai Medical College, Fudan University Shanghai China; ^3^ Department of Gynecologic Oncology Fudan University Shanghai Cancer Center Shanghai China

**Keywords:** gastric cancer, GSPT1, Hsa_circ_0001944, miR‐498

## Abstract

**Background:**

Gastric cancer (GC) is the fifth most common malignant tumor and the third leading cause of cancer‐related deaths worldwide. CircRNAs may provide new insights into the development of GC by acting as oncogenes or tumor suppressors. In this study, we aim to examine the biological role of hsa_circ_0001944 (circFIRRE) in tumor progression of GC.

**Methods:**

The bioinformatic analysis, qPCR, Western blotting, and immunohistochemistry were fulfilled to detect the expression of hsa_circ_0001944, miR‐498, and GSPT1 in gastric cancer. Gain or loss of function approaches were used to investigate the biological functions of hsa_circ_0001944. MTS, EDU, wound healing, and transwell assays were performed to study the proliferation, invasion, and migration of GC cells. These molecular mechanisms were detected by luciferase reporter assays and chromatin immunoprecipitation assays.

**Results:**

We screened out hsa_circ_0001944, whose expression was significantly increased in gastric cancer tissues. Knockdown of hsa_circ_0001944 significantly suppressed the cell proliferation, invasion, and migration. Mechanistic investigations showed that hsa_circ_0001944 can bind to and sponge miR‐498. Moreover, hsa_circ_0001944 sponged miR‐498 to increase GSPT1 expression, thereby promoted excessive proliferation and maintained the malignant phenotype of GC cells.

**Conclusion:**

The present study demonstrates the hsa_circ_0001944/miR‐498/GSPT1 axis contributes to GC development. This may provide a target for GC therapy and potential prognostic biomarker.

## Graphical abstract

We aim to examine the oncogenic role of CircFIRRE in gastric cancer. Knockdown of circFIRRE suppressed cell proliferation, invasion and migration. Moreover, circFIRRE sponged miR‐498 to increase GSPT1 expression, thereby promoted the malignant phenotype of gastric cancer. The present study demonstrates the circFIRRE/miR‐498/GSPT1 axis contributes to gastric cancer development.

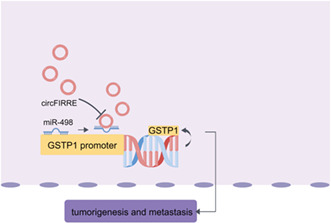

## 1 INTRODUCTION

As the fifth most common malignancy, gastric cancer (GC) is the third major cause of cancer‐associated death worldwide.^1^ There are many patients diagnosed with GC in advanced phase with poor prognosis.^2^ Although progress has been realized in treating GC recently, modest benefit can be achieved. All these concerns show an emergent demand for identification of mechanisms promoting gastric cancer development.

Notably, growing evidences have demonstrated that non‐coding RNAs exerted essential effects on the tumorigenesis, metastasis, and chemoradiotherapy resistance of gastric cancer.^3^ Different from linear RNAs, circular RNAs (circRNAs) mean a sort of closed circular RNA molecules that are widely found in the human body and have no 3′ or 5′ ends.^4^ As oncogenes or tumor suppressors, circRNAs make contributions to tumor appearance and growth.^5^, ^6^ Hsa_circ_0001944 (CircFIRRE), a novel circRNAs, has been reported in non‐small cell lung cancer and makes contributions to glycolysis and tumor growth via miR‐142‐5p/NFAT5.^7^ However, there is no reported research on its role in gastric cancer, which needs further research.

It is reported that circRNAs perform their molecular function through several mechanisms. MiRNA sponging acts as the most widely reported mechanism of circRNAs' roles in developing cancers.^8^, ^9^, ^10^ In our study, we found that hsa_circ_0001944 could bind to and sponge miR‐498 in gastric cancer cells.

G1 to S phase transition 1 (GSPT1) has been demonstrated oncogenic activity in gastric cancer.^11^ GSPT1 was the targeted gene of miR‐144 or miR‐27b‐3p in gastric cancer.^12^ MiRNA has been reported to induce targeted mRNA's degradation or translational repression by binding to its 3'‐UTR.^13^, ^14^, ^15^ The current research forecast that GSPT1 has the putative binding sites with miR‐498, which was certificated in the further study.

The current research observed that hsa_circ_0001944 was overexpressed in gastric cancer and can drive the malignant phenotype of gastric cancer cells. Hsa_circ_0001944 could regulate the expression of GSPT1 via miR‐498 sponging in gastric cancer. Therefore, our study found that the hsa_circ_0001944/miR‐498/GSPT1 axis can regulate the development of gastric cancer and may also give a hidden target for gastric cancer treatment.

## 2 RESULTS

### 2.1 Hsa_circ_0001944 over‐expressed in gastric cancer tissues

**FIGURE 1:**
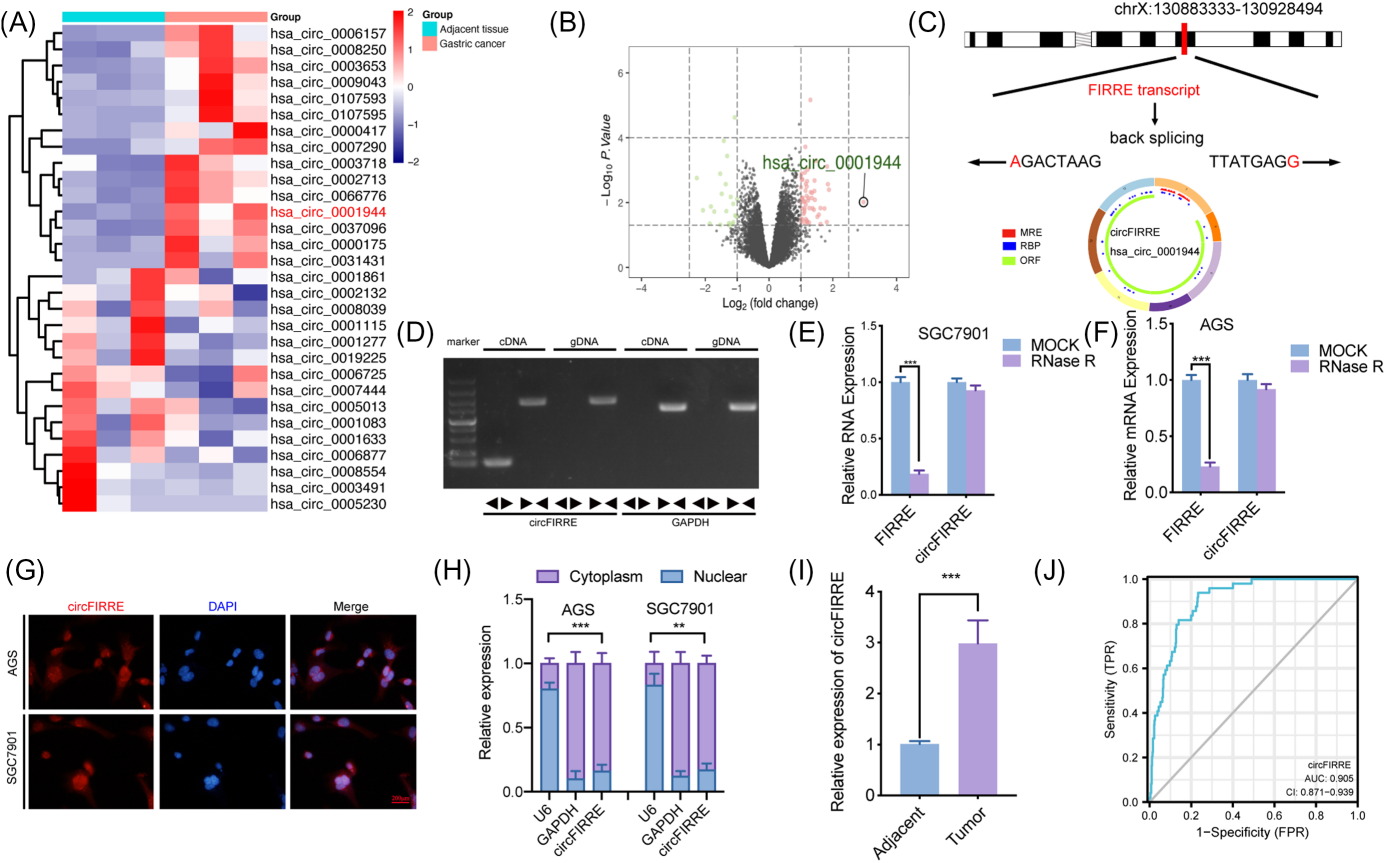
(A)Hsa_circ_0001944 over‐expressed in gastric cancer tissues. a, (b) The up‐regulation of hsa_circ_0001944 expression was found in gastric cancer tissues by comparing with nearby normal tissues via GSE121445. (C) Schematic description of hsa_circ_0001944 generation by circularizing exons in the FIRRE gene, and Sanger sequencing detected the head‐to‐tail splicing of hsa_circ_0001944. (D) Agarose gel electrophoresis of qPCR on hsa_circ_0001944 expression via divergent and convergent primers. (E)GAPDH was used as a linear control. (F) qPCR with or without RNase R. (SGC7901: *p* < 0.001; AGS: *p* < 0.001; Student's *t* test) was adopted to detect the expression of hsa_circ_0001944 and FIRRE in both SGC7901 and AGS. (G) FISH assays showing the cellular localization of hsa_circ_0001944 in gastric cancer cells. (H) Nuclear‐cytoplasmic fractionation assay showed hsa_circ_0001944 was predominantly localized in the cytoplasm. (I) The expression of hsa_circ_0001944 was up‐regulated in gastric cancer tissues than the nearby normal control detected by qPCR. (Adjacent vs. Tumor: *n* = 40, *p* < 0.001, Student's *t* test). (J) ROC curves of hsa_circ_0001944 in gastric cancer. All data were expressed as the mean ± SD (three independent experiments). **p < 0.01; ***p < 0.001.

Firstly, we analyzed the Gene Expression Omnibus (GEO) dataset GSE121445 and observed hsa_circ_0001944 was the highest up‐regulated circRNAs in gastric cancers compared with adjacent normal tissues (Figure 1A,B). Hsa_circ_0001944 (circFIRRE, chrX:130883333–130,928,494) was back‐spliced from the transcript of FIRRE mRNA (Figure 1C). In order to demonstrate its circular structure, we designed divergent and convergent primers to study whether cDNA or gDNA could amplify hsa_circ_0001944 respectively. The gel electrophoresis results showed that hsa_circ_0001944 was amplified only in cDNA and produced via reverse splicing (Figure 1D). Then, we performed RNase R assays and the results showed a slight expression change of hsa_circ_0001944 after RNase R treatment, while its linear FIRRE decreased significantly (Figure 1E,F). FISH assays showed hsa_circ_0001944 was primarily localized in the cytoplasm of gastric cancer cells (Figure 1G). Besides, we performed nuclear–cytoplasm separation assays, and the results showed hsa_circ_0001944 was predominantly localized in the cytoplasm (Figure 1H), indicating miRNA sponge may be one of the main mechanisms of circFIRRE. To verify the expression difference in hsa_circ_0001944 in the adjacent normal tissues and gastric cancer tissues, we furtherly detected it in forty collected paired samples, and observed that the expression of hsa_circ_0001944 was up‐regulated in gastric cancers compared with the nearby normal tissues (Figure 1I). Analysis of ROC curves demonstrated that hsa_circ_0001944 may be a potential biomarker of gastric cancer (AUC = 90.5%) (Figure 1J). In addition, the correlation between hsa_circ_0001944 expression with clinicopathological features was shown in Table S1, high hsa_circ_0001944 expression was correlated with poor prognostic factors (including poor differentiation and advanced TNM stage).

### 2.2 Hsa_circ_0001944 knockdown inhibited the proliferation and invasion of gastric cancer cells

**FIGURE 2:**
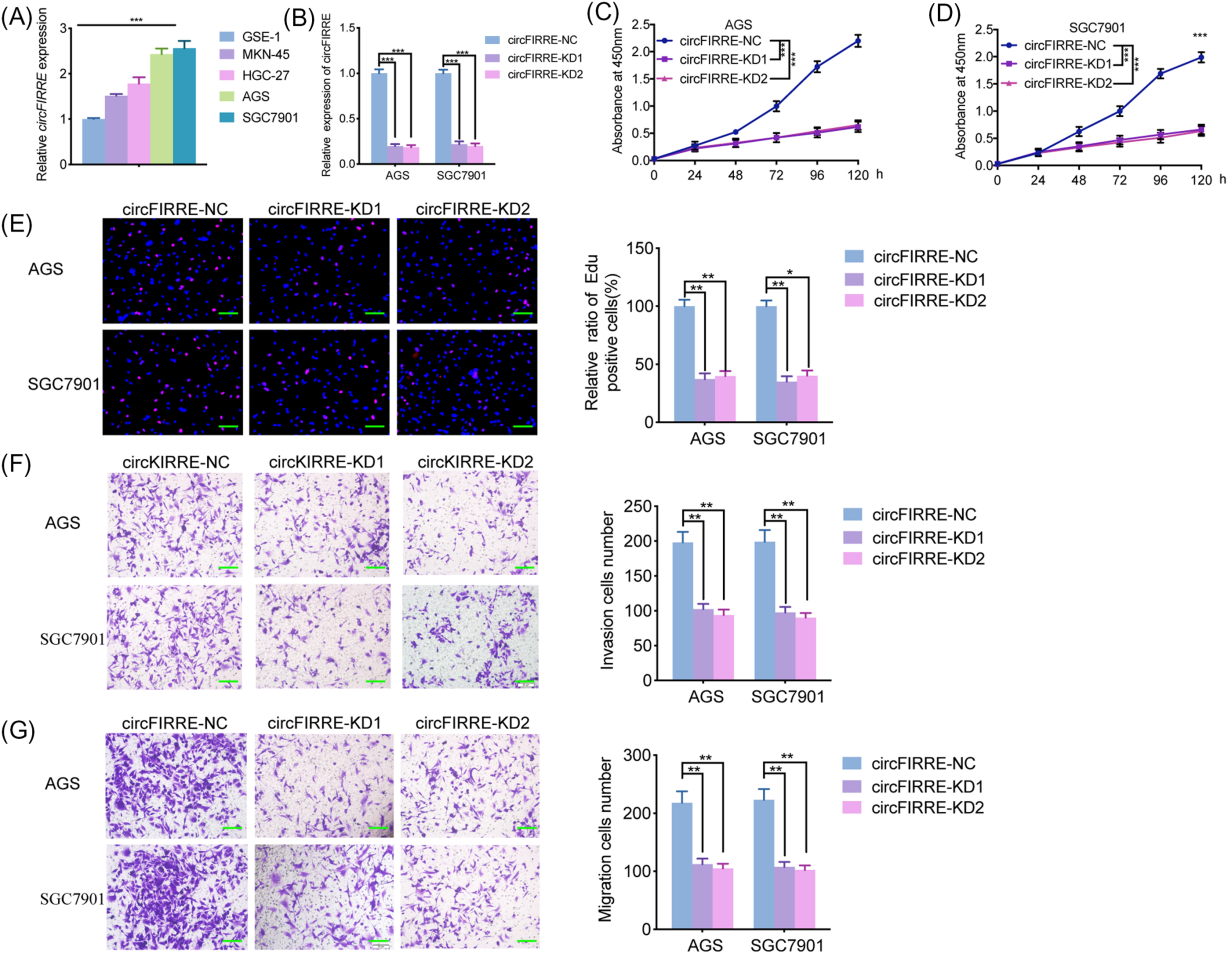
Hsa_circ_0001944 knockdown inhibited the proliferation and invasion of gastric cancer cells. a: The expression of hsa_circ_0001944 in varied gastric cancer cell lines was detected by qPCR. b: The qPCR validated the efficiency of hsa_circ_0001944 knockdown in SGC7901 and AGS. c, d: MTS assays displayed that hsa_circ_0001944 knockdown suppressed the cell viability of SGC7901 and AGS. (SGC7901: *p* < 0.001; AGS: *p* < 0.001; one‐way ANOVA). e: EDU assays displayed that hsa_circ_0001944 knockdown suppressed the propagation of SGC7901 and AGS. Scale bar = 100 μm. (SGC7901: *p* < 0.05; AGS: *p* < 0.01; one‐way ANOVA). f, g: Transwell (f) and migration assays (g) showed the aggression and migration of SGC7901 and AGS were suppressed after hsa_circ_0001944 knockdown. Scale bar = 50 μm. All data were expressed as the mean ± SD (three independent experiments). *p < 0.05; **p < 0.01; ***p < 0.001.

Expression of hsa_circ_0001944 in different gastric cancer cell lines was evaluated by qPCR, AGS, and SGC7901 cell lines showed a relatively higher expression (Figure 2A). Thus, these two cell lines were chosen as candidates for hsa_circ_0001944 knockdown in vitro, and qPCR also showed the efficiency of hsa_circ_0001944 knockdown (Figure 2B). We furtherly confirmed that the cell viability, propagation, aggression, and migration of AGS and SGC7901 were suppressed after hsa_circ_0001944 knockdown via MTS, EDU, transwell, and migration assays (Figure 2C–G). Moreover, we also studied whether hsa_circ_0001944 overexpression could exert the opposite effects on gastric cancer cells. After hsa_circ_0001944 expression was up‐regulated in AGS and SGC7901 (Figure S1A), all the MTS, Edu, transwell, and migration assays showed hsa_circ_0001944 overexpression obviously promoted the cell viability, proliferation, invasion, and migration of gastric cancer cell (Figure S1B–I). Taken together, hsa_circ_0001944 knockdown inhibited the proliferation and invasion of gastric cancer cells.

### 2.3 Hsa_circ_0001944 could bind to and sponge miR‐498 in gastric cancer cells

**FIGURE 3:**
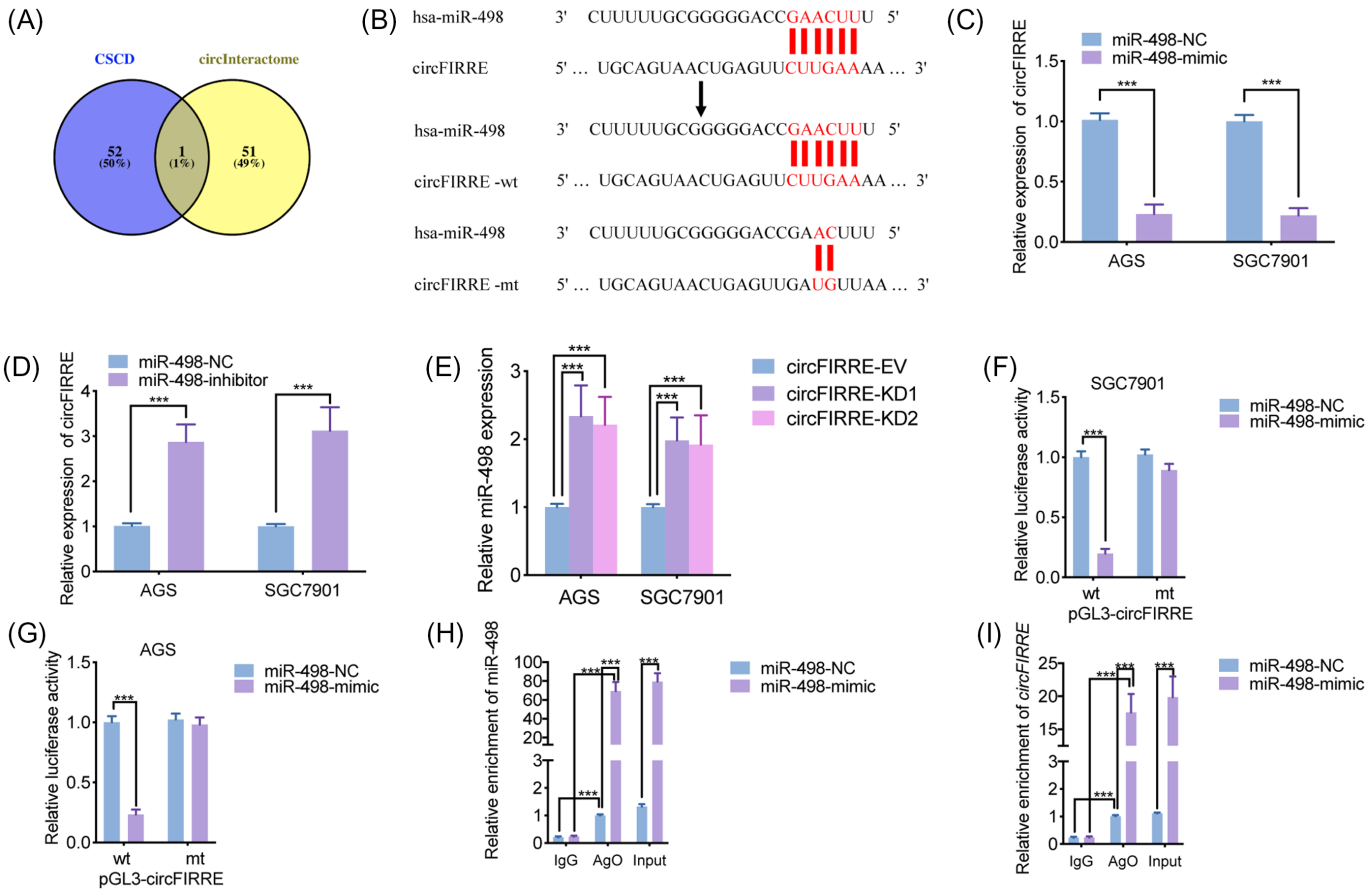
Hsa_circ_0001944 can bind to and sponge miR‐498 in gastric cancer cells. a: The hidden targeted miRNAs of hsa_circ_0001944 were forecast based on CSCD and circInteractome databases. b: The accurate binding site for hsa_circ_0001944 and miR‐498. c: qPCR displayed that the expression of hsa_circ_0001944 was downregulated in SGC7901 and AGS after miR‐498 mimic treatment. (SGC7901: *p* < 0.001; AGS: *p* < 0.001; Student's *t* test). d: qPCR showed that the expression of hsa_circ_0001944 was up‐regulated in SGC7901 and AGS after miR‐498 inhibitor treatment. (SGC7901: *p* < 0.001; AGS: *p* < 0.001; Student's *t* test). e: qPCR displayed that the expression of miR‐498 was enhanced in SGC7901 and AGS after hsa_circ_0001944 knockdown. (SGC7901: *p* < 0.01; AGS: *p* < 0.01; one‐way ANOVA). f, g: According to the luciferase reporter assays, miR‐498 mimic downregulated the luciferase activities of hsa_circ_0001944 in SGC7901 and AGS. (SGC7901: *p* < 0.001; AGS: *p* < 0.001; Student's *t* test). h, i: The RIP assays were made in SGC7901 (h) and AGS (i) after transfecting the miR‐498 mimic, then enrichment of hsa_circ_0001944 and miR‐498 was examined. (*p* < 0.001; One‐Way ANOVA). All data were expressed as the mean ± SD (three independent experiments). *p < 0.05; **p < 0.01; ***p < 0.001.

Since miRNA sponging is one of the leading mechanisms of circRNAs in the growth of several tumors, we furtherly predicted the potential target miRNAs of hsa_circ_0001944 based on CSCD and circInteractome databases. We observed that miR‐498 was the only candidate miRNAs for hsa_circ_0001944 in both CSCD and circInteractome (Figure 3A). The bindings site for miR‐498 on hsa_circ_0001944 was shown in Figure 3B. Then, qPCR displayed that the expression of hsa_circ_0001944 was declined in miR‐498 mimic‐processed AGS and SGC7901 but grew in miR‐498 inhibitor‐processed cells (Figure 3C,D). It was also confirmed that the expression of miR‐498 enhanced in AGS and SGC7901 after hsa_circ_0001944 knockdown (Figure 3E). To confirm that miR‐498 can bind to hsa_circ_0001944 directly, luciferase reporter plasmids were constructed with wild‐type hsa_circ_0001944 and mutant‐type hsa_circ_0001944 (Figure 3B). We furtherly confirmed that the luciferase activity of the hsa_circ_0001944‐wt vector greatly declined in miR‐498 mimic‐processed SGC7901 and AGS. Nevertheless, the luciferase activity of the hsa_circ_0001944‐mt vector had no great differences (Figure 3F,G). Furthermore, we performed the RIP assay and found significant enrichment of hsa_circ_0001944 and miR‐498 in AGS and SGC7901 after miR‐498 mimic treatment (Figure 3H,I). In summary, hsa_circ_0001944 could bind to and sponge miR‐498 in gastric cancer cells.

### 2.4 Hsa_circ_0001944 knockdown induced cell proliferation and invasion suppression via miR‐498

**FIGURE 4:**
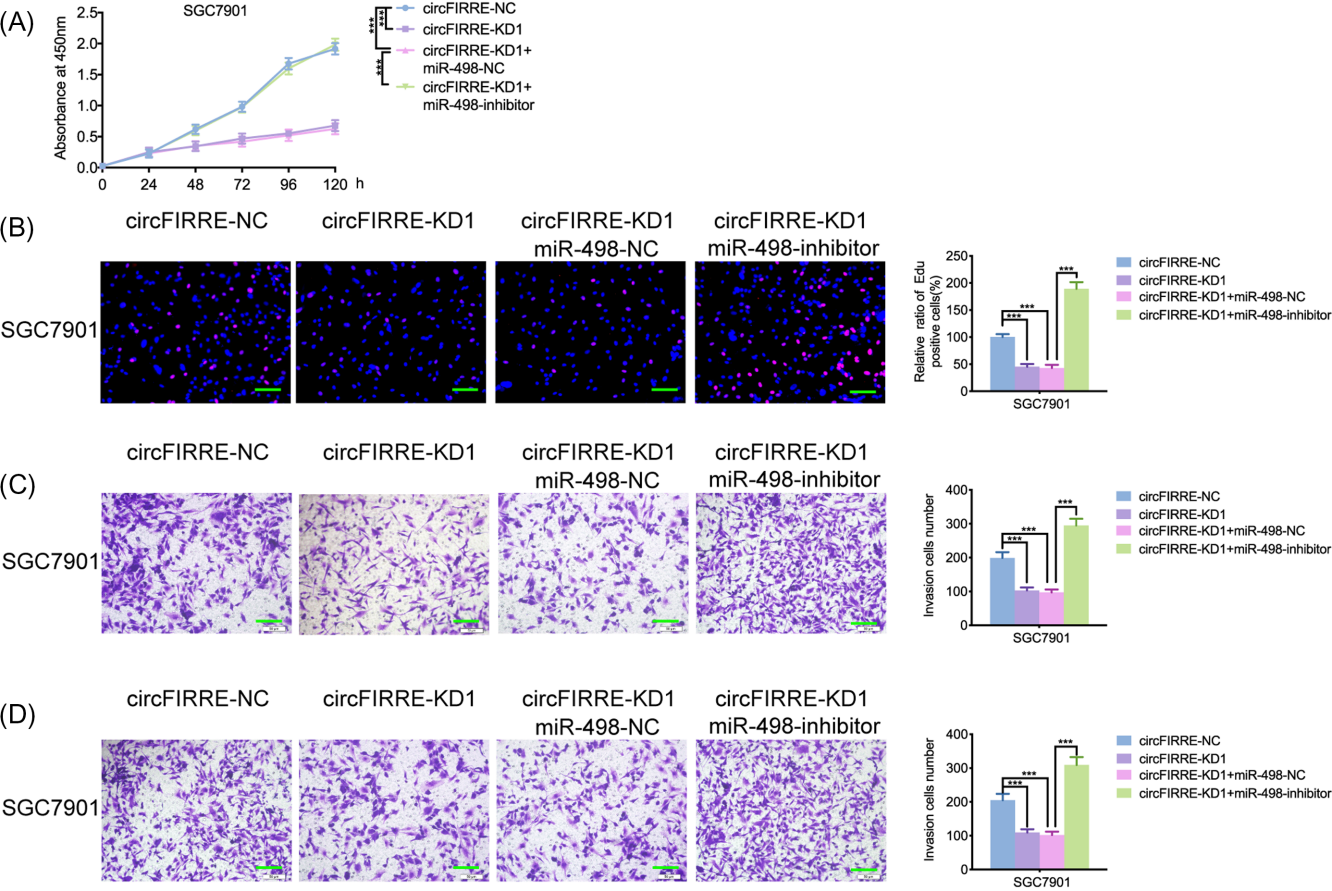
Knockdown of hsa_circ_0001944 inhibited cell propagation and invasion by recovery of the function of miR‐498. a: MTS assays displayed that hsa_circ_0001944 knockdown decreased the cell viability of SGC7901 but reversed after miR‐498 inhibitor treatment. (*p* < 0.001; One‐Way ANOVA). b: The EDU assays displayed that hsa_circ_0001944 knockdown decreased the propagation of SGC7901 but reversed after miR‐498 inhibitor treatment. Scale bar = 100 μm. (*p* < 0.001; one‐way ANOVA). c, d: Transwell (c) and migration assays (d) displayed the aggression and migration of SGC7901 decreased after hsa_circ_0001944 knockdown but reversed after miR‐498 inhibitor treatment. Scale bar = 50 μm. (*p* < 0.001; one‐way ANOVA). All data were expressed as the mean ± SD (three independent experiments). *p < 0.05; **p < 0.01; ***p < 0.001.

All the MTS, EDU, transwell, and migration assays confirmed that knockdown of hsa_circ_0001944 inhibited the cell viability, propagation, aggression, and migration of SGC7901. However, these inhibiting effects were reversed after miR‐498 inhibition (Figure 4A–D). In conclusion, knockdown of hsa_circ_0001944 inhibited the propagation and aggression of gastric cancer cells by recovery of the function of miR‐498.

### 2.5 Hsa_circ_0001944 sponges miR‐498 and regulates GSPT1 expression in gastric cancer cells

**FIGURE 5:**
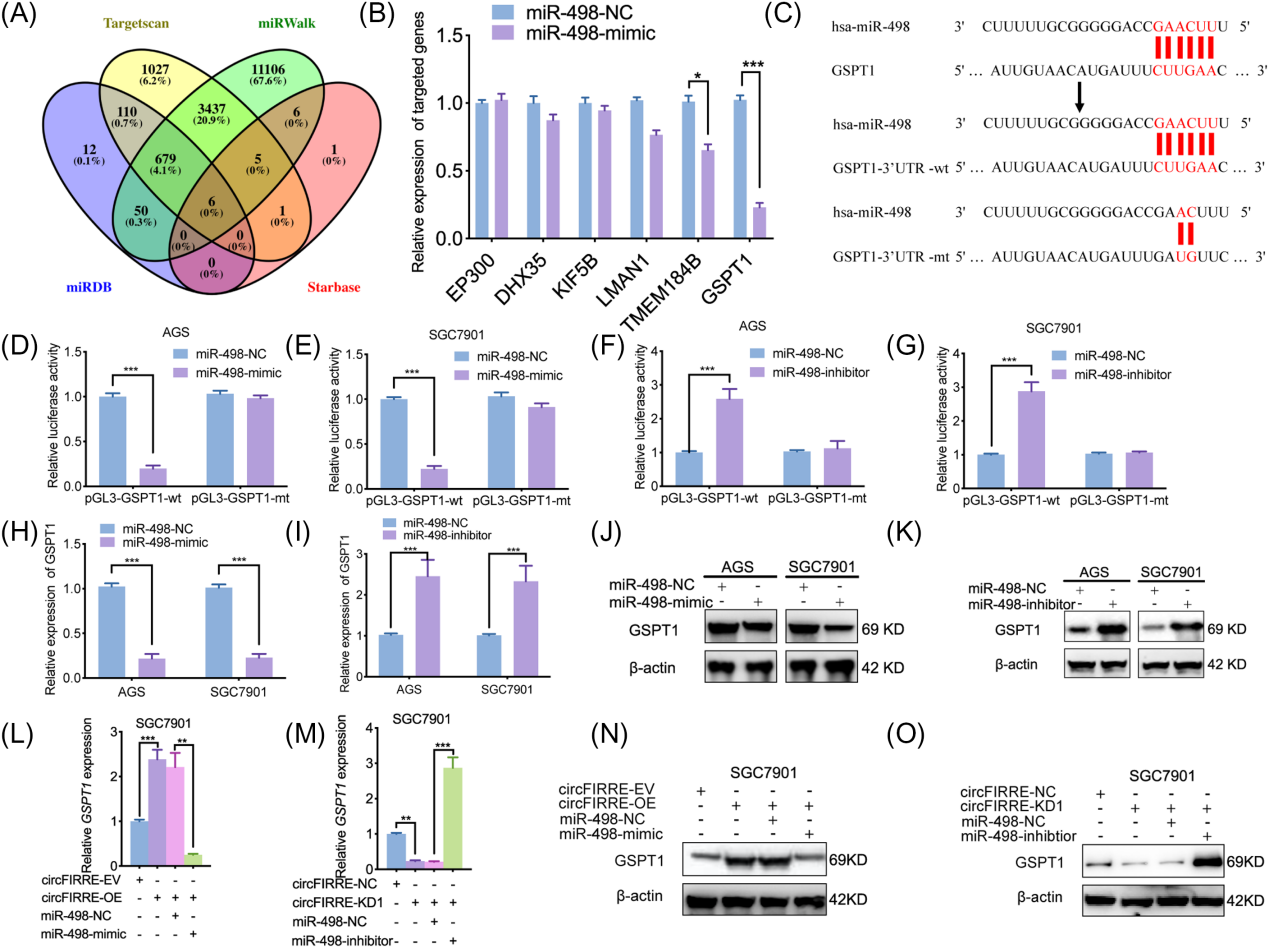
Hsa_circ_0001944 sponges miR‐498 and regulates GSPT1 expression in gastric cancer cells. a: Identification of six miRNAs potentially regulating GSPT1 expression on basis of TargetScan, miRWalk, miRDB, and Starbase databases. b: Schematic diagram of the putative miR‐498 binding site in the 3′‐UTR of GSPT1. c: qPCR displayed the expression of 6 candidate mRNAs after miR‐498 mimic treatment in SGC7901. d‐g: According to the luciferase reporter assays, miR‐498 mimic (d, e) or inhibitor (f, g) regulate the luciferase activities of GSPT1 in AGS and SGC7901. (AGS: *p* < 0.001; SGC7901: *p* < 0.001; Student's t test). h–k: qPCR (h, j) and Western blotting (i, k) displayed that GSPT1 expression in AGS and SGC7901 after miR‐498 mimic or inhibitor treatment. (AGS: *p* < 0.001; SGC7901: *p* < 0.001; Student's *t* test). l‐o: qPCR (l, m) and Western blotting (n, o) displayed that GSPT1 expression in SGC7901 after hsa_circ_0001944 overexpression or knockdown followed with miR‐498 mimic or inhibitor treatment. (*p* < 0.01; One‐Way ANOVA). All data were expressed as the mean ± SD (three independent experiments). *p < 0.05; **p < 0.01; ***p < 0.001.

As described above, hsa_circ_0001944 could bind to and sponge miR‐498 in gastric cancer cells. Thus, according to TargetScan, miRDB, miRWalk, and Starbase databases, the bidding of miR‐498 to the 3′‐UTR of EP300, DHX35, KIF5B, LMAN1, TMEM184B, and GSPT1 (Figure 5A) could be forecast. In order to find the candidate mRNA of miR‐498, we performed qPCR to detect these 6 mRNAs expression in SGC7901 under miR‐498 mimic treatment and found GSPT1 was the most significantly down‐regulated mRNA (Figure 5B). Actually, GSPT1 has been reported as promoting gastric cancer cell proliferation, invasion, and migration.^12^ Thus, luciferase reporter assays and luciferase reporter plasmids with wild‐type and mutant forms of the GSPT1 3'‐UTR we constructed (Figure 5C) were made. The relative luciferase activity of the GSPT1‐wt vector was greatly declined in miR‐498 mimic‐processed AGS and SGC7901 cells (Figure 5D,E). Nevertheless, the opposite results were obtained in miR‐498 inhibitor‐treated AGS and SGC7901 cells (Figure 5F,G). Besides, the expression of GSPT1 grew after the miR‐498 inhibitor treatment via qPCR and Western blotting (Figure 5I,K), while down‐regulated after miR‐498 mimic treatment (Figure 5H,J). Moreover, expression of GSPT1 increased in SGC7901 cells after hsa_circ_0001944 overexpression and these effects were reversed by miR‐498 mimic treatment (Figure 5L,N), while the opposite results were also observed in hsa_circ_0001944 knockdown and/or miR‐498 inhibitor treatment (Figure 5M,O). In summary, hsa_circ_0001944 sponges miR‐498 and furtherly regulates the expression of GSPT1 in gastric cancer cells.

### 2.6 GSPT1 overexpression attenuated hsa_circ_0001944 knockdown‐induced cell proliferation and invasion suppression

**FIGURE 6:**
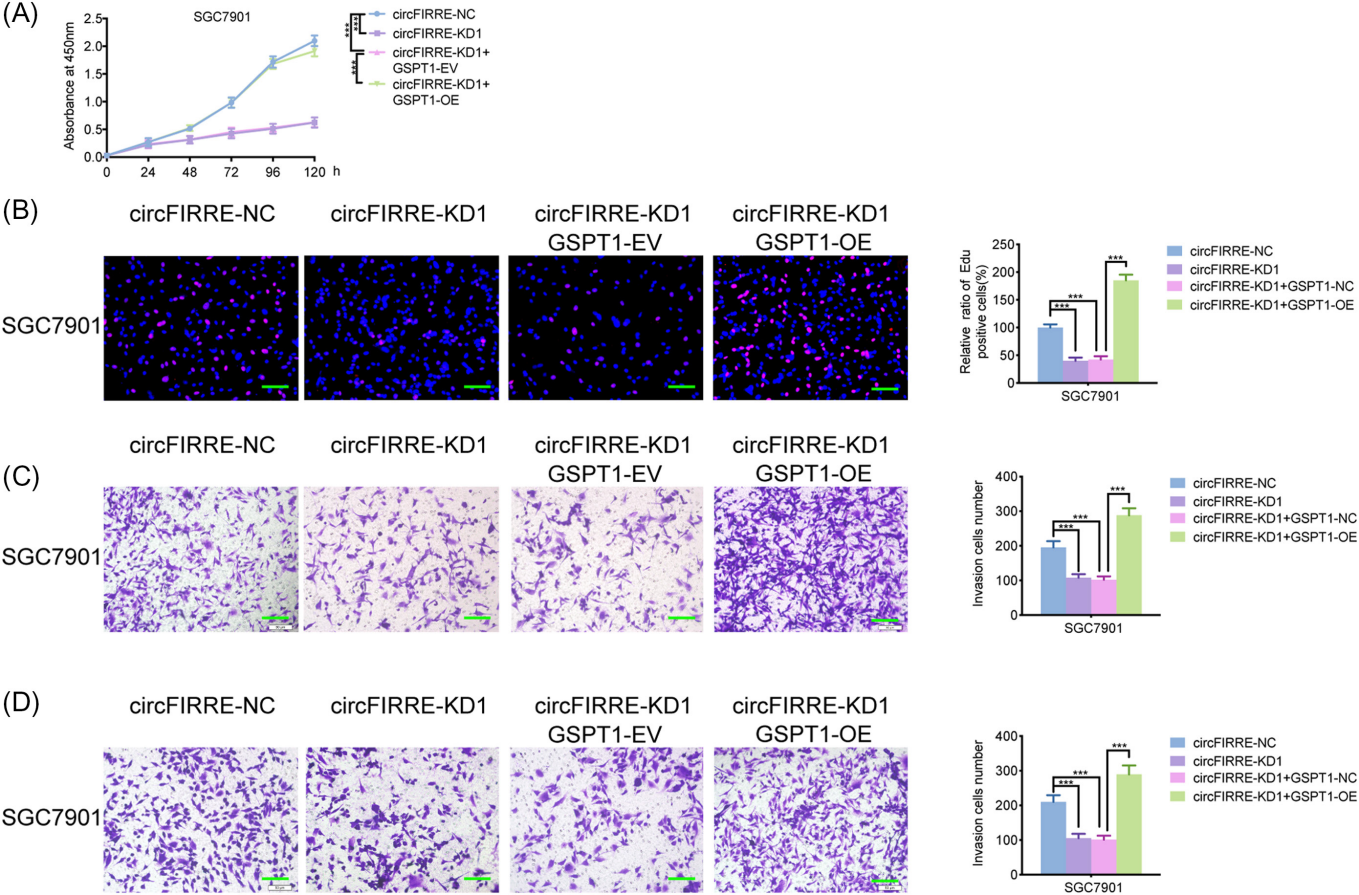
GSPT1 overexpression attenuated hsa_circ_0001944 knockdown‐induced cell proliferation and invasion suppression. a: MTS assays showed that the inhibition effect of circFIRRE‐knockdown in cell viability was reversed by GSPT1 overexpression. (*p* < 0.001; one‐way ANOVA). b: The EDU assays showed that the inhibition effect of circFIRRE knockdown in cell proliferation was reversed by GSPT1 overexpression. Scale bar = 100 μm. (*p* < 0.001; one‐way ANOVA). c, d: The inhibition effects of circFIRRE‐knockdown in cell invasion (c) and migration (d) were reversed by GSPT1 overexpression. Scale bar = 50 μm. (*p* < 0.001; one‐way ANOVA). All data were expressed as the mean ± SD (three independent experiments). *p < 0.05; **p < 0.01; ***p < 0.001.

As reported above, hsa_circ_0001944 knockdown inhibited cell viability, proliferation, invasion, and migration of SGC7901. However, these inhibiting effects were reversed under GSPT1 overexpression (Figure 6A–D). Therefore, we can conclude that hsa_circ_0001944 promoted cell propagation and invasion via GSPT1.

### 2.7 Knockdown of hsa_circ_0001944 suppressed tumorigenesis of gastric cancer in vivo

**FIGURE 7:**
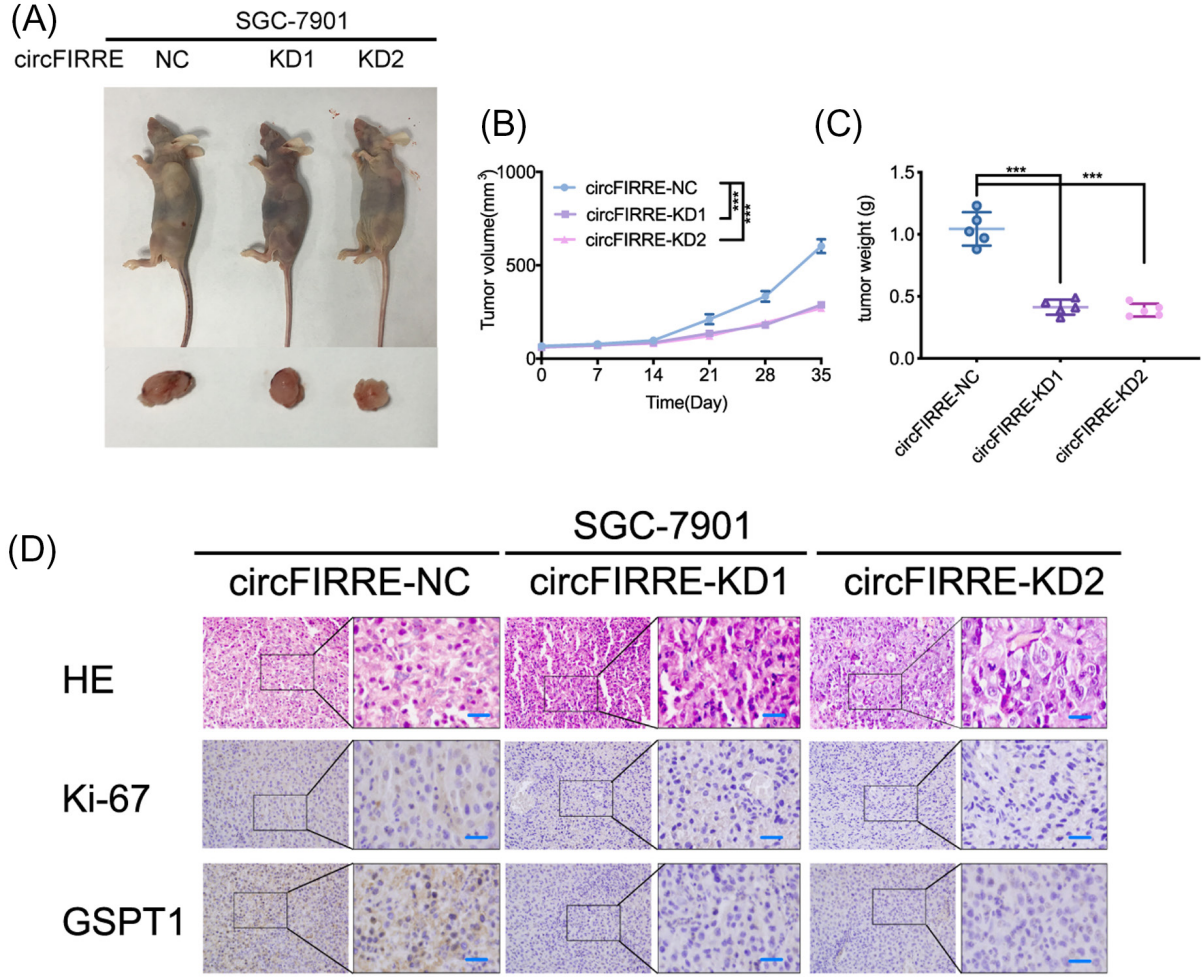
Knockdown of hsa_circ_0001944 suppressed tumorigenesis of gastric cancer in vivo. a: Images of subcutaneous xenograft tumors of SGC7901 cells. b, c: The measured tumor volumes (b) and tumor weights (c) were decreased under hsa_circ_0001944 knockdown. (*p* < 0.001; one‐way ANOVA). d: Representative immunohistochemical staining showed the expression of Ki67 and GSPT1 in the control and hsa_circ_0001944 knockdown models. Scale bar = 50 μm. All data were expressed as the mean ± SD (three independent experiments). *p < 0.05; **p < 0.01; ***p < 0.001.

Thereafter, orthotopic xenografts were made to decide the role of hsa_circ_0001944 knockdown in vivo. Both tumor volumes and tumor weights were reduced after hsa_circ_0001944 knockdown (Figure 7A–C). Immunohistochemistry displayed declined expression of Ki67 and GSPT1 in the hsa_circ_0001944 knockdown group (Figure 7D). Therefore, hsa_circ_0001944 knockdown suppressed tumorigenesis of gastric cancer in vivo.

## 3 DISCUSSION

Gastric cancer is one of the most life‐threatening malignancies worldwide.^16^ Previous studies have elucidated that most cancers are genetic diseases. Differential display code 3, also referred to as PCA3, was agreed by the Food and Drug Administration (FDA) for prostate cancer diagnosis as the first case of a lncRNA implied for clinical test by FDA approval.^17^, ^18^ A comparative study has certified that the incidence of gastric cancer is associated with the expression changes in corresponding RNA and protein in cancer cells.^19^ Growing evidence proves that lncRNAs exert significant effects on GC carcinogenesis, proliferation, and metastasis,^20^ but the most featured biomarker is still unknown.

CircRNAs are closed‐loop RNAs abundant in various tissues of the human body and can be divided into noncoding and coding circRNAs.^21^ For example, circβ‐catenin aggravates the malignant phenotype of non‐small‐cell lung cancer via encoding a peptide.^22^ CircRNAs show a circular structure which is difficult to be broken down compared with mRNAs.^23^ Previous studies have confirmed that circRNAs could affect tumor progress. For example, hsa_circ_001988 has a low expression in colorectal cancer and correlates with clinical significance,^24^ overexpression of circCHAF1A promoted propagation and tumorigenesis by MDM2‐dependent p53 signaling in glioma.^25^ In gastric cancer, circDLG1 boosted gastric cancer progress and anti‐PD‐1 resistance via miR‐141‐3p/CXCL12.^26^

This research firstly confirmed increased expression of hsa_circ_0001944 in gastric cancer tissues. As reported, circ_0001944 contributed to glycolysis and tumor development by up‐regulating NFAT5 as a decoy for miR‐142‐5p in non‐small cell lung cancer.^7^ It also promoted brain metastasis by regulating miR‐125a/BRD4 axis in breast cancer.^27^ It also drove the development and metastasis of bladder cancer as a competitive endogenous RNA for miR‐548.^28^ Since those studies demonstrated that hsa_circ_0001944 exerted a vital effect on the development of several types of cancers, it is reasonable to raise the issue whether hsa_circ_0001944 has similar functions in gastric cancer. Subsequently, we confirmed that hsa_circ_0001944 knockdown inhibited the cell viability, propagation, aggression, and migration of gastric cancer cells AGS and SGC7901 in vitro. These outcomes demonstrated that hsa_circ_0001944 acted as an oncogene in gastric cancer.

CircRNAs functions in various mechanisms, such as RBP sponge, miRNA sponge, directly regulating mRNA transcription, or translation. For instance, the expression of circRNA_100269 is reduced in gastric cancer, and it can suppress the development of tumor cells by targeting miR‐630 (31). MiRNA sponging is one of the most important molecular mechanisms of circRNAs. This research proved that hsa_circ_0001944 could bind to and sponge miR‐498 in gastric cancer cells, and the inhibiting effects of hsa_circ_0001944 knockdown could all be reversed by miR‐498 inhibitor treatment.

As previously described, circRNAs can regulate mRNA expression via miRNA sponging. Our study forecasted that miR‐498 could bind to the 3′‐UTR of GSPT1. GSPT1 acts as an oncogene in gastric cancer. Therefore, GSPT1 could be a reasonable downstream gene of hsa_circ_0001944 to exert its cancer‐promoting effect. Meanwhile, we confirmed that hsa_circ_0001944 sponged miR‐498 and subsequently regulated GSPT1 expression in gastric cancer cells. GSPT1 overexpression also reversed the inhibiting effects of hsa_circ_0001944 knockdown. Meanwhile, the same conclusion in vivo was reached. Therefore, our research demonstrated that hsa_circ_0001944 enhanced GSPT1 expression via sponging miR‐498 to promote gastric cancer cell proliferation and invasion.

The current research demonstrates the circFIRRE/miR‐498/GSPT1 axis contributes to gastric cancer development, which may offer a target for gastric cancer therapy and potential prognostic biomarker.

## 4 METHODS

### 4.1 Patient samples and ethical approval

Shanghai Cancer Center, Fudan University offered forty paired samples from gastric cancer patients and the nearby normal tissues. The detailed clinical information was listed in Table S1. This research got the consent of the ethics committee of Shanghai Cancer Center, Fudan University, and each patient had written informed consent.

### 4.2 Cell culture

Offered by the Chinese Academy of Sciences cell bank, the human gastric cancer cell line AGS and SGC7901 were cultured in RPMI1640 (FBS; Gibco), added with 1% penicillin/streptomycin (Gibco), and 10% fetal bovine serum (FBS) at 37°C with 5% CO_2_.

### 4.3 Lentiviral vector establishment and transfection

The lentivirus transfection and effect identifications were made based on the manufacturer's guidance. Gene‐Chem was adopted for constructing the vectors based on lentivirus for the overexpression of hsa_circ_0001944 and GSPT1, RNAi‐regulated knockdown of hsa_circ_0001944, and their negative regulation. The miR‐498 inhibitor (Assay ID: MH34359; Thermo Fisher Scientific), mimic (Assay ID: MC34359, Thermo Fisher Scientific), and Thermo Fisher Scientific (Assay ID: 4464076) offered the negative controls. qPCR was used to detect the transfection efficacy. Table S2 lists the sequences of siRNAs.

### 4.4 RNA extraction, RNase R treatment, nuclear‐cytoplasmic fraction, and qPCR

The qPCR was made based on the manufacturer's guidance. To be brief, the total RNA of gastric cancer tissues and cell lines was extracted by the Mini‐BEST Universal RNA Extraction kit (TaKaRa). In terms of circRNA and mRNA, the reserve transcription of RNA was made into cDNA via the Prime Script RT Master Mix reagent kit (TaKaRa). With regard to miRNA, the Prime Script™ RT reagent kit (TaKaRa) was adopted for synthesizing cDNA. Then, the SYBR Green Master Mix (TaKaRa) with PCR LightCycler480 (Roche Diagnostics) was employed to make the qPCR assays. The TaqMan Universal Master Mix II (Applied Biosystems) was adopted for detecting the expression of miR‐498. Thermo Fisher Scientific (Primer ID: Hs00502366_CE) offered the primers of miR‐498. Table S3 lists all of the relative primers. Moreover, RNase R (Epicentre Technologies) was performed for certifying hsa_circ_0001944 and remove the role of linear FIRRE RNA. β‐actin was adopted as the internal regulation for circRNA, miRNA, or mRNA.

### 4.5 Western blotting

A total cell protein extraction kit (KeyGen Biotechnology) was adopted for separating total proteins of gastric cancer tissues or cells. Next, following SDS‐polyacrylamide gel electrophoresis (SDS‐PAGE), the transfer of total protein for every sample onto the polyvinylidene difluoride (PVDF) membranes was conducted. Next, 2% bovine serum albumin (KeyGen Biotechnology) was adopted to block PVDF membranes at room temperature for 2 h, and the overnight incubation of all these membranes was made at 4°C with the primary antibodies below: GSPT1 (1:1000; #ab126090, Abcam) and β‐actin (1:200; #ab115777, Abcam). After 2 h of secondary antibody incubation, the detection of all the bands was made with a chemiluminescence ECL kit (Beyotime Biotechnology), and all the bands were quantified with the ImageJ software (National Institutes of Health, Bethesda). The calculation of the expression of proteins was made on basis of the internal regulation β‐actin.

### 4.6 Immunohistochemistry (IHC)

Embedded in paraffin, gastric cancer tissues were sliced into 4 mm sections and labeled with primary antibodies as follows: Ki67 (1:100; #ab92742, Abcam), GSPT1 (1:100; #ab126090, Abcam). Then, after the treatment with an immunohistochemical labeling kit (MaxVision Biotechnology), the slices were imaged via a light microscope (Olympus). According to the German immunohistochemical mark, the expression degrees and the staining intensity were evaluated.^29^

### 4.7 Luciferase reporter assay

Luciferase reporter assays were made based on the manufacturer's instructions. Briefly, Gene‐Chem was adopted for constructing all the luciferase reporter plasmids (hsa_circ_0001944‐wt and hsa_circ_0001944‐mt, GSPT1‐wt and GSPT1‐mt). Then, after being seeded into 96‐well plates at a density of 1 × 10^4^ cells/well, the transfection of gastric cancer cell line AGS and SGC7901 with relative luciferase reporter plasmids was made, followed by 48‐hour relative treatment. In the end, relative luciferase activities were evaluated via the Dual‐Luciferase Reporter Assay System (Promega) and calculated as the proportion of firefly luciferase activity to Renilla luciferase activity.

### 4.8 RNA immunoprecipitation (RIP) assay

Based on the producer's guidance, the RIP assays were made via an Imprint RNA Immunoprecipitation Kit (Sigma). Firstly, the lysis of all gastric cancer cell line AGS and SGC7901 under the varied conditions was made in RIP buffer, such as magnetic beads conjugated with the negative control IgG and anti‐AGO2 (Millipore). Then, the immunoprecipitated RNAs were obtained after incubation with Proteinase K buffer (Omega). In the end, the detection of precipitants was made with the qPCR assays.

### 4.9 Cell viability assay

Cell viability assays were made based on the manufacturer's guidance. Briefly, the seeding of gastric cancer cell line AGS and SGC7901 into 96‐well plates was made at a density of 1 × 10^3^ cells/well, followed by incubation for 0, 24, 48, 72, 96, and 120 h. Then, according to the manufacturer's guidance, the detection of cell viability under different conditions was made by the CellTiter 96® Aqueous Non‐Radioactive Cell Proliferation Assay Kit (Promega).

### 4.10 EDU assay

EDU assays were made based on the producer's guidance. Briefly, the EDU assays were adopted for detecting the proliferation of gastric cancer cell line AGS and SGC7901 via the EDU assay kit (Beyotime, Biotechnology) based on the producer's guidance. Firstly, the seeding of AGS and SGC7901 cells into 24‐well plates was made at 1 × 10^5^ cells/well for 24 h. Next, 10 μM EDU reagent was put to the medium, followed by 2‐hour incubation at 37°C with 5% CO2. After fixture with 4% paraformaldehyde (solarbio) and permeabilization with 0.5% Triton ×‐100 (solarbio), the AGS, and SGC cells were counterstained. In the end, a laser scanning confocal microscope (Olympus) was adopted to evaluate the percentage of EDU‐positive cells.

### 4.11 Xenograft experiments

Based on the Animal Care Committee of Shanghai Cancer Center, Fudan University, xenograft experiments were made as illustrated.^30^ First of all, the Six‐week‐old female BALB/c nude mice (Beijing Vital River Laboratory Animal Technology) were fallen into the hsa_circ_0001944‐NC group, the hsa_circ_0001944‐KD1 group, and the hsa_circ_0001944‐KD2 group. Under specific pathogen‐free conditions, every group with five mice was bred in the Laboratory Animal Center of Shanghai Cancer Center, Fudan University. The injection of gastric cancer cell SGC7901 treated with different conditions into the back flanks of the nude mouse (5 × 10^4^ cells per mouse) was made. The daily observation of every group was conducted for no distress or death sign, followed by sacrificed mice and isolated whole tumor. Finally, tumor volume and tumor weight were calculated. A Vernier caliper detected the tumor size, and the formula below: V = (D × d)/2 mm was adopted to calculate the tumor volume, where D means the longest diameter and d refers to the shortest diameter of the tumor.

### 4.12 Bioinformatics analysis

The basic information of hsa_circ_0001944 was acquired from circBase (http://www.circbase.org/). Cancer‐Specific circRNA database (CSCD, http://gb.whu.edu.cn/CSCD/) and CircInteractome (https://circinteractome.nia.nih.gov) databases were adopted for predicting the sponging miRNAs of circFIRRE. MiRDB (http://mirdb.org), TargetScan (www. targetscan.org), miRWalk (http://mirwalk.umm. uni‐heidelberg.de), and Starbase (http://starbase.sysu.edu.cn) were adopted for predicting possible the targeting mRNAs of miR‐498.

### 4.13 Statistical analysis

All experiments were made at least three times, and all of the outcomes were shown as the mean ± SD, and SPSS 25.0 software (SPSS, Chicago, IL, USA) was adopted to make the statistical analysis. The two‐tailed Student's *t* test and the chi‐square test were employed to evaluate the comparisons of two independent groups. One‐way analysis of variance evaluated the statistical significance among three or more groups. Pearson's correlation analysis evaluated the correlation between two groups. Two‐tailed p Values <0.05 was of significance.

## FUNDING INFORMATION

This work was supported by the National Natural Science Foundation of China 82203752.

## CONFLICT OF INTEREST

The authors disclose no potential conflicts of interest.

## Supporting information


Figure S1.

Table S1.

Table S2.

Table S3.

## Data Availability

The data that support the findings of this study are available on request from the corresponding author. The data are not publicly available due to privacy or ethical restrictions.
