# Supplementary figures and images for: Hsa_circ_0001944 enhanced GSPT1 expression via sponging miR‐498 to promote proliferation and invasion of gastric cancer
Source: J Clin Lab Anal. 2023 Jan 4;37(2):e24810. doi: 10.1002/jcla.24810 (PMC9937881; doi:10.1002/jcla.24810)

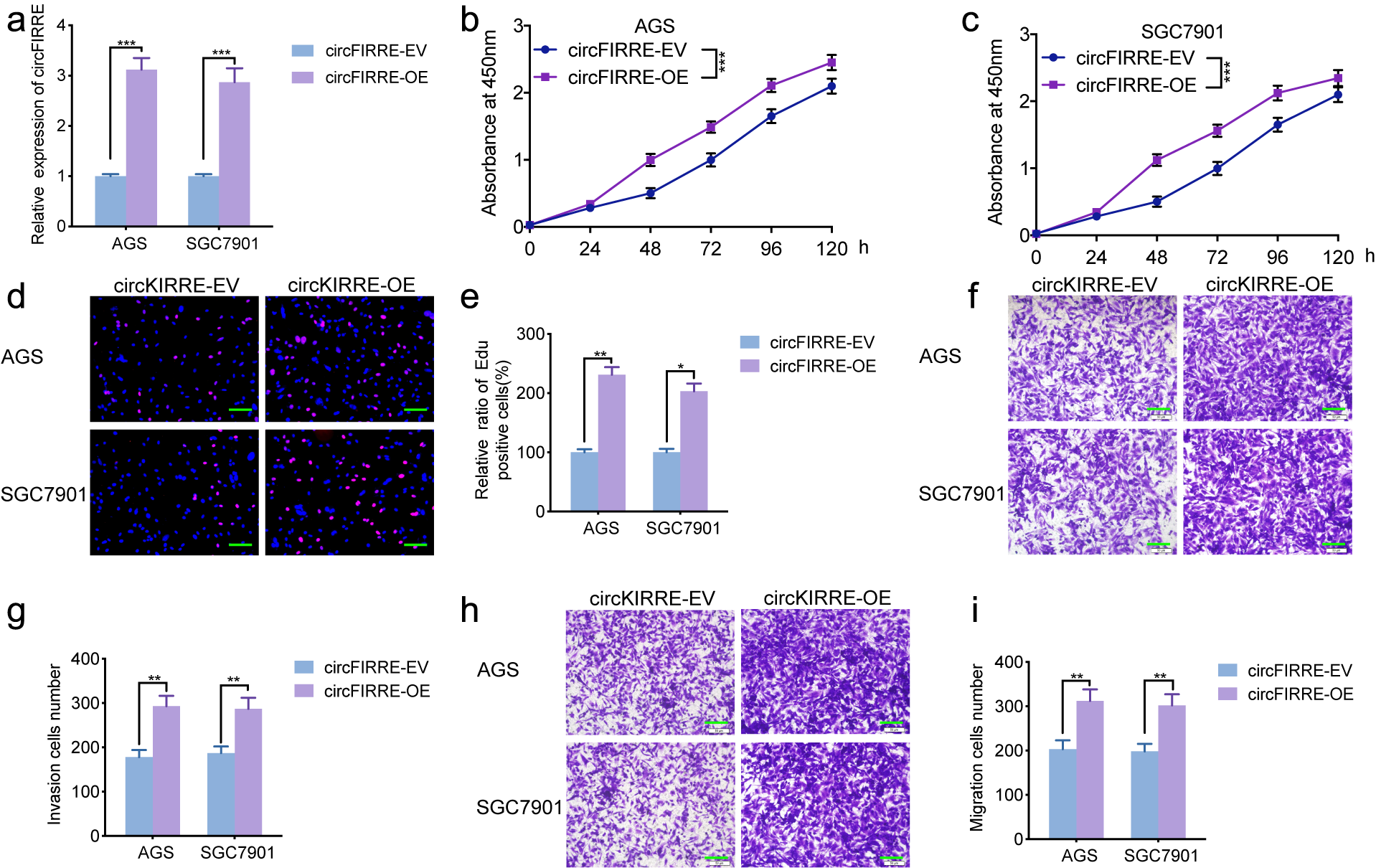

Supplement: Supplementary file 1 — Figure S1. [file JCLA-37-e24810-s003.tif]
