# Supplementary material for: Hsa_circ_0001944 enhanced GSPT1 expression via sponging miR‐498 to promote proliferation and invasion of gastric cancer
Source: J Clin Lab Anal. 2023 Jan 4;37(2):e24810. doi: 10.1002/jcla.24810 (PMC9937881; doi:10.1002/jcla.24810)
Supplement: Supplementary file 2 — Table S1. [file JCLA-37-e24810-s002.docx]

**Supplementary table 1 Correlation between circFIRRE expression and clinicopathological parameters in gastric cancer (n = 40)**

| Parameters | Category | No. | circFIRRE expression* | | χ2 | p |
| --- | --- | --- | --- | --- | --- | --- |
|  |  |  | Low (20) | High (20) |  |  |
| Age |  |  |  |  |  |  |
|  | <65 | 25 | 13 | 12 | 0.106 | 0.744 |
|  | ≥65 | 15 | 7 | 8 |  |  |
| Gender |  |  |  |  |  |  |
|  | Male | 24 | 11 | 13 | 0.417 | 0.519 |
|  | Female | 16 | 9 | 7 |  |  |
| Differentiation |  |  |  |  |  |  |
|  | Well | 7 | 6 | 1 | 4.329 | 0.037 |
|  | Moderate+ Poor | 33 | 14 | 19 |  |  |
| T stage |  |  |  |  |  |  |
|  | T1+T2 | 15 | 11 | 4 | 5.227 | 0.022 |
|  | T3+T4 | 25 | 9 | 16 |  |  |
| N stage |  |  |  |  |  |  |
|  | N0+N1 | 17 | 12 | 5 | 5.013 | 0.025 |
|  | N2+N3 | 23 | 8 | 15 |  |  |
| M stage |  |  |  |  |  |  |
|  | M0 | 33 | 19 | 14 | 4.329 | 0.038 |
|  | M1 | 7 | 1 | 6 |  |  |

*: CircFIRRE expression was detected by qRT-PCR and ranked from low to high. The high expression of CircFIRRE was defined as the expression level higher than the median expression level of CircFIRRE.
