# Supplementary material for: Hsa_circ_0001944 enhanced GSPT1 expression via sponging miR‐498 to promote proliferation and invasion of gastric cancer
Source: J Clin Lab Anal. 2023 Jan 4;37(2):e24810. doi: 10.1002/jcla.24810 (PMC9937881; doi:10.1002/jcla.24810)
Supplement: Supplementary file 3 — Table S2. [file JCLA-37-e24810-s004.doc]

**Table S2. siRNA and miRNA sequences**

| **Primer** | **Forward (5’-3’)** | **Reverse (5’-3’)** |
| --- | --- | --- |
| circFIRRE-KD1 | UCUUUAAGAACUCAGUUUCUG | GAAACUGAGUUCUUAAAGAGA |
| circFIRRE-KD2 | UGUAGUUUCAGCUUCUUGGAA | CCAAGAAGCUGAAACUACAAG |
| siRNA-NC | UUCUUCGAAGGUGUCACGUTT | ACGUGACACCUUCGAAGAATT |

Stem-loop Sequence of miR498:

AACCCUCCUUGGGAAGUGAAGCUCAGGCUGUGAUUUCAAGCCAGGGGGCGUUUUUCUAUAACUGGAUGAAAAGCACCUCCAGAGCUUGAAGCUCACAGUUUGAGAGCAAUCGUCUAAGGAAGUU

miR498 mimic sequence:

AAAGCACCUCCAGAGCUUGAAGCMapped
