# Supplementary material for: Hsa_circ_0001944 enhanced GSPT1 expression via sponging miR‐498 to promote proliferation and invasion of gastric cancer
Source: J Clin Lab Anal. 2023 Jan 4;37(2):e24810. doi: 10.1002/jcla.24810 (PMC9937881; doi:10.1002/jcla.24810)
Supplement: Supplementary file 4 — Table S3. [file JCLA-37-e24810-s001.doc]

**Table S3. PCR Primers**

**q**PCR Primers

| **Primer** | **Forward (5’-3’)** | **Reverse (5’-3’)** |
| --- | --- | --- |
| circFIRRE | AAACTGCCGAACAACCAAAG | ACCACCCCTTTTTGGGTATC |
| GSPT1 | TCAGGACTTACTGGAGCAAATCT | AACTTCCACGTTGTGCTTGTT |
| miR-498 | TGTAAAACGACGGCCAGT | CAGGAAACAGCTATGACC |
| β-actin | CATGTACGTTGCTATCCAGGC | CTCCTTAATGTCACGCACGAT |
